# Regional disparities in suicide among patients with cancer: A nationwide population‐based study in Japan

**DOI:** 10.1002/cam4.6574

**Published:** 2023-09-22

**Authors:** Ken Kurisu, Saki Harashima, Maiko Fujimori, Tatsuo Akechi, Kazuhiro Yoshiuchi, Yosuke Uchitomi

**Affiliations:** ^1^ Division of Survivorship Research National Cancer Center Institute for Cancer Control Tokyo Japan; ^2^ Department of Stress Sciences and Psychosomatic Medicine, Graduate School of Medicine The University of Tokyo Tokyo Japan; ^3^ Department of Psychiatry and Cognitive‐Behavioral Medicine Nagoya City University, Graduate School of Medical Sciences Nagoya Japan; ^4^ Innovation Center for Supportive, Palliative and Psychosocial Care, National Cancer Center Tokyo Japan

**Keywords:** cancer, geographical inequality, prefecture, regional disparities, suicide

## Abstract

**Background:**

This study aimed to explore prefecture‐level differences in suicide risk among patients with cancer in Japan.

**Methods:**

Data from the National Cancer Registry, which covers the entire Japanese population, were used. Patients diagnosed with cancer between 2016 and 2017 were included. The standardized mortality ratio (SMR) for suicide within 2 years after cancer diagnosis was quantified compared with the general population for each prefecture. Multivariate Poisson regression analysis was conducted to quantify the adjusted relative risk using Hokkaido as the reference.

**Results:**

The analysis included 2,133,502 patients. The SMRs were high among patients with cancer residing in certain prefectures, such as the Hokuriku region (the middle and western parts of Japan's main island). These areas also exhibited a significant relative risk in the Poisson regression model.

**Conclusion:**

The results demonstrated that patients with cancer in certain prefectures in Japan have a high suicide risk.

## 1 INTRODUCTION

Patients with cancer are reportedly at an increased risk of committing suicide.^1^, ^2^, ^3^, ^4^ Inspired by these global reports, we previously conducted the first nationwide study in Japan to investigate the risk of suicide following cancer diagnosis compared with the general population using the National Cancer Registry (NCR).^5^, ^6^ These studies revealed that the suicide risk was higher in patients with cancer than in the general population,^5^ and this result was also observed even 2 years after diagnosis,^6^ with a particularly elevated risk among patients with advanced disease immediately after diagnosis.^6^

Japan, characterized by high suicide rates among developed nations,^7^ has responded to this issue by enacting the Basic Act for Suicide Prevention.^8^ In the general Japanese population, the variability in suicide rates across prefectures has received substantial attention, and widespread research has revealed many suicide‐related factors.^9^, ^10^, ^11^, ^12^, ^13^, ^14^, ^15^, ^16^, ^17^ In contrast, no nationwide research has examined such variations among patients with cancer in Japan. The Fourth Basic Plan to Promote Cancer Control Programs, proposed by the Ministry of Health, Labour and Welfare of Japan, necessitates tailored measures considering the unique circumstances of each local community.^18^ Thus, investigating regional disparities is warranted to advance countermeasures for suicide prevention among patients with cancer.

Since our previous study,^6^ more data have been accumulated in the NCR database, resulting in sufficient sample sizes to analyze regional variation. Therefore, this study aimed to investigate regional variations in the risk of suicide following cancer diagnosis using the NCR. We hypothesized that substantial differences in suicide risk would exist across prefectures among patients with cancer.

## 2 MATERIALS AND METHODS

### 2.1 Study participants and measurements

This study employed the NCR, which covers the entire Japanese population.^19^ Records of patients diagnosed with cancer between January 1, 2016 and December 31, 2017 were extracted. Patients diagnosed through autopsy or death certificate, those whose addresses were outside Japan, and those with unknown age, sex, address or time of diagnosis were excluded.

The study outcome was suicide occurring during the 2 years following a cancer diagnosis, as categorized by the International Classification of Diseases, 10th Edition, codes X60–X84 and Y87.0. The NCR contains the patient addresses at the prefectural level, enabling us to quantify suicide rates within each prefecture.

### 2.2 Prefecture‐level measurements

To investigate the factors contributing to regional differences, items at the prefecture level were analyzed. We selected the factors potentially associated with suicide rates in patients with cancer that were obtainable, such as alcohol consumption sourced from the Comprehensive Survey of Living Conditions^20^ and reimbursement points for palliative care from the NDB Open Data^21^ (Table S1).

### 2.3 Statistical analysis

We quantified the standardized mortality ratio (SMR) of suicide in patients with cancer compared with the general Japanese population. The SMR is the ratio of the observed suicide counts among patients with cancer to the expected count in the general population. The observed counts were obtained from the NCR. The expected count was calculated by multiplying the observation period for each patient by the suicide rate in the general Japanese population with the corresponding age, sex, observation year, and residential prefecture. The inclusion of residential prefectures enabled adjustment for regional differences in suicide rates among the general population. The suicide rates of the general population were derived from the Vital Statistics of Japan provided by the Ministry of Health, Labour and Welfare and Population Estimates. Patients registered with zero months of survival in the NCR were assigned a value of 0.5 months. Byar's method was used to estimate 95% confidence intervals (CIs) for the SMRs.

Multivariate Poisson regression analysis was performed to examine prefectural variations in suicide rates. To quantify the relative risks of the SMRs, we added an offset of the log of the expected number of deaths. We transformed the NCR dataset, which originally contained individual data with a binary outcome, into a clustered dataset, compiled according to the extent of tumors, primary tumor site, and prefecture. Each cluster included total suicide count as an outcome. The extent of tumors and certain primary tumor sites were reportedly significant predictors of suicide in patients with cancer.^6^, ^22^ Therefore, these variables were incorporated into the multivariate model.

The correlation coefficient was quantified between the SMR of suicide and prefecture‐level variables.

All the analyses were conducted using R (version 4.3.1) with the package “epiR” (version 2.0.63) and “NipponMap” (version 0.2). Statistical significance was set at *p* < 0.05.

## 3 RESULTS

### 3.1 Patient characteristics

This study included 2,133,502 patients, comprising 1,074,738 and 1,058,764 patients diagnosed in 2016 and 2017, respectively. Table S2 presents the descriptive statistics. Within 2 years following diagnosis, 1307 suicides were observed, 658 of which were among patients diagnosed in 2016 and 649 among those diagnosed in 2017. The overall SMR was 1.86 (95% CI, 1.76–1.97), which was nearly identical to that in our previous study that solely included patients diagnosed in 2016.^6^

### 3.2 Prefectural profile of SMR

**FIGURE 1:**
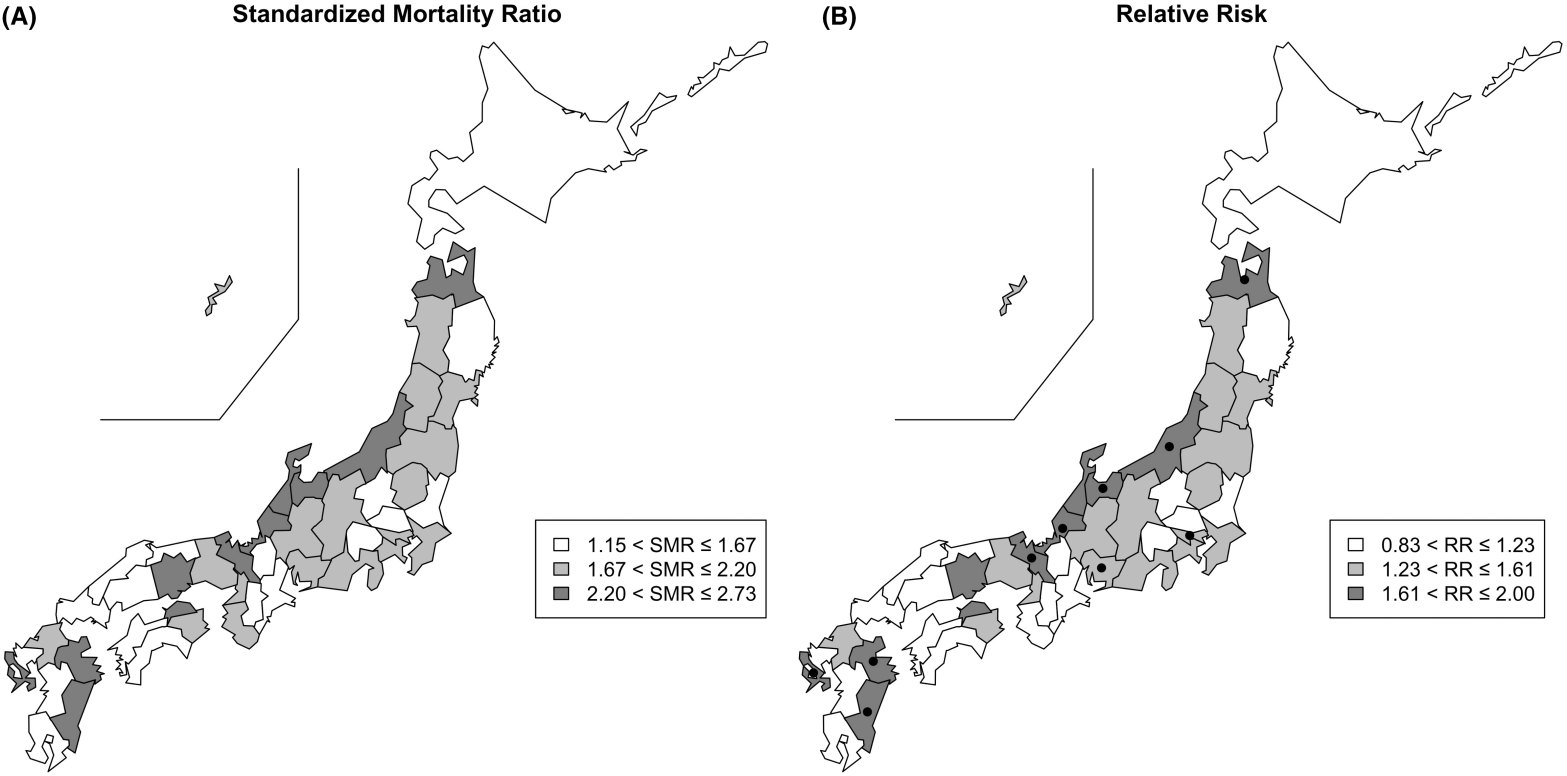
Maps of (A) standardized mortality ratio (SMR) for suicide and (B) relative risk (RR) in the Poisson regression model. The range from the maximum to the minimum value was divided into three intervals of approximately equal length, that is, 1.53 for (A) and 0.39 for (B), and prefectures within each numerical interval were color‐coded for visualization. In map (B), dots indicate statistical significance when compared with Hokkaido as a reference.

Figure 1A depicts the map of Japan indicating the SMR across each prefecture, while Table 1 (left) shows the values, along with the raw and expected suicide rates. These rates were calculated by dividing the observed and expected suicide counts by the number of patients with cancer in each prefecture (see Figure S1 for the distribution).

**TABLE 1:** Standardized mortality ratio and adjusted relative risk of suicide within 2 years following cancer diagnosis.

Prefecture	SMR (95% CI) [Raw suicide rates/expected suicide rates]	Relative risk (95% CI)
Hokkaido	1.39 (1.02–1.86)* [4.40/3.16]	1.00 (Reference)
Aomori	2.73 (1.85–3.88)* [11.93/4.37]	2.00 (1.27–3.16)*
Iwate	1.29 (0.66–2.25) [5.36/4.17]	0.94 (0.50–1.78)
Miyagi	2.07 (1.33–3.08)* [6.16/2.97]	1.52 (0.93–2.50)
Akita	1.96 (1.19–3.02)* [9.12/4.66]	1.41 (0.84–2.39)
Yamagata	1.69 (0.92–2.83) [6.52/3.87]	1.26 (0.69–2.29)
Fukushima	1.89 (1.21–2.81)* [7.11/3.76]	1.41 (0.86–2.31)
Ibaraki	1.61 (1.04–2.38)* [5.28/3.28]	1.17 (0.72–1.91)
Tochigi	2.20 (1.39–3.30)* [7.40/3.37]	1.60 (0.97–2.64)
Gunma	1.40 (0.80–2.27) [4.96/3.55]	1.03 (0.58–1.81)
Saitama	1.64 (1.25–2.12)* [5.24/3.20]	1.20 (0.81–1.76)
Chiba	1.92 (1.47–2.46)* [6.15/3.20]	1.41 (0.96–2.06)
Tokyo	2.15 (1.80–2.56)* [6.58/3.06]	1.58 (1.13–2.21)*
Kanagawa	1.90 (1.51–2.37)* [5.65/2.97]	1.40 (0.98–2.01)
Niigata	2.37 (1.73–3.17)* [9.91/4.18]	1.73 (1.15–2.61)*
Toyama	2.59 (1.53–4.09)* [8.94/3.45]	1.90 (1.10–3.28)*
Ishikawa	2.39 (1.34–3.94)* [7.48/3.13]	1.76 (0.98–3.15)
Fukui	2.63 (1.31–4.70)* [8.04/3.06]	1.93 (1.00–3.73)*
Yamanashi	1.44 (0.58–2.97) [5.00/3.47]	1.06 (0.48–2.35)
Nagano	1.92 (1.17–2.97)* [5.44/2.83]	1.44 (0.85–2.44)
Gifu	1.92 (1.22–2.88)* [6.74/3.51]	1.40 (0.85–2.31)
Shizuoka	1.79 (1.23–2.51)* [5.49/3.07]	1.30 (0.83–2.03)
Aichi	2.06 (1.59–2.62)* [6.04/2.94]	1.49 (1.02–2.18)*
Mie	1.62 (0.93–2.63) [5.23/3.23]	1.18 (0.67–2.09)
Shiga	1.36 (0.62–2.59) [3.93/2.88]	0.99 (0.49–2.03)
Kyoto	2.39 (1.61–3.41)* [6.41/2.68]	1.76 (1.11–2.78)*
Osaka	1.77 (1.44–2.17)* [6.35/3.58]	1.29 (0.91–1.83)
Hyogo	1.71 (1.29–2.22)* [5.68/3.33]	1.24 (0.84–1.84)
Nara	1.60 (0.82–2.79) [4.86/3.05]	1.20 (0.63–2.26)
Wakayama	1.68 (0.87–2.93) [6.55/3.91]	1.21 (0.64–2.28)
Tottori	1.26 (0.34–3.22) [3.64/2.89]	0.92 (0.33–2.55)
Shimane	1.15 (0.42–2.50) [4.21/3.67]	0.84 (0.36–1.96)
Okayama	2.28 (1.41–3.48)* [6.37/2.79]	1.67 (1.00–2.80)
Hiroshima	1.59 (1.05–2.32)* [5.31/3.33]	1.18 (0.74–1.91)
Yamaguchi	1.36 (0.70–2.37) [4.53/3.34]	1.00 (0.53–1.88)
Tokushima	1.74 (0.70–3.58) [5.23/3.02]	1.26 (0.57–2.79)
Kagawa	2.40 (1.31–4.03)* [7.57/3.15]	1.79 (0.98–3.25)
Ehime	1.29 (0.67–2.26) [4.50/3.48]	0.95 (0.50–1.79)
Kochi	1.21 (0.44–2.63) [4.20/3.47]	0.88 (0.38–2.06)
Fukuoka	1.88 (1.42–2.46)* [6.14/3.26]	1.36 (0.92–2.02)
Saga	1.48 (0.59–3.05) [4.58/3.10]	1.07 (0.48–2.37)
Nagasaki	2.36 (1.46–3.61)* [7.42/3.14]	1.75 (1.04–2.93)*
Kumamoto	1.60 (0.91–2.59) [5.04/3.15]	1.15 (0.65–2.03)
Oita	2.61 (1.57–4.08)* [9.22/3.53]	1.91 (1.12–3.27)*
Miyazaki	2.61 (1.57–4.08)* [10.19/3.90]	1.89 (1.11–3.23)*
Kagoshima	1.28 (0.70–2.15) [4.92/3.83]	0.94 (0.51–1.70)
Okinawa	2.10 (1.17–3.46)* [8.13/3.88]	1.54 (0.86–2.76)

*Note*: Raw and expected suicide rates are shown as incidences per 10,000 patients. Relative risk was quantified using the Poisson regression model, adjusting for the extent of tumors and primary tumor sites.

Abbreviations: CI, confidence interval; SMR, standardized mortality ratio.

* *p* < 0.05.

Prefectures with elevated SMRs were predominantly located in the Hokuriku region, consisting of Niigata, Toyama, Ishikawa, and Fukui prefectures. The SMR ranged from 1.15 (95% CI, 0.42–2.50) in Shimane prefecture to 2.73 (95% CI, 1.85–3.88) in Aomori prefecture.

### 3.3 Prefectural profile of relative risks

Figure 1B and Table 1 (right) show the relative risks in the Poisson regression model, which was adjusted by the extent of tumors and primary tumor sites. Hokkaido was chosen as the reference merely because it is the first prefecture in the official numbering of prefectures by the Ministry of Health, Labour and Welfare; additionally, Hokkaido has a relatively large sample size both for suicide count and the entire population, making it a suitable reference.

The distribution was similar to that of the SMRs, ranging from 0.84 (95% CI, 0.36–1.96) in Shimane prefecture to 2.00 (95% CI, 1.27–3.16) in Aomori prefecture. Ten prefectures showed significantly elevated relative risks. The unadjusted relative risks were similar to the adjusted values (Table S3).

### 3.4 Correlation between SMR and prefecture‐level measurements

No items measured at the prefecture‐level revealed a significant correlation with the suicide SMR. Further details are provided in Table S4.

## 4 DISCUSSION

This is the first population‐based study in Japan to reveal the prefectural profile of suicide risk among patients with cancer. Patients residing in certain prefectures had a significantly higher risk of suicide.

The Hokuriku region showed a high relative risk of suicide among patients with cancer. This geographical concentration may indicate the existence of social stigma toward cancer or cultural influences in these regions. Previous studies have investigated the stigma toward cancer^23^ and validated a tool to quantify it.^24^ Moreover, cultural influences on suicide are pertinent issues in Japan.^25^ Additional investigations are needed to reveal the potential effects of stigma and cultural factors in these regions.

The SMR and relative risk warrant careful interpretation as they were based on both the suicide rates of patients with cancer and those of the general population. For instance, Aomori prefecture demonstrated high suicide rates among both patients with cancer and the general population. Okayama prefecture also showed a high SMR; however, its suicide rates were relatively low among both groups. In areas such as Aomori, suicide prevention strategies for both groups may be required. Conversely, Iwate prefecture, which showed a low SMR due to a high suicide rate in the general population, may require countermeasures primarily targeted at the general population.

A study using data from the United States identified several states with high suicide risk among patients with cancer, which may be attributed to certain ethnic communities with elevated suicide risk within these states.^26^ In contrast, Japan is a relatively monoethnic nation, making such an explanation inapplicable. Another investigation involving 635 counties in the United States revealed significant associations between suicide risk among patients with cancer and county‐level income and urban/rural status.^27^ However, we were unable to detect an association between suicide and prefecture‐level income or categorize the regions as urban or rural. Further detailed investigations may enable the detection of similar associations in Japan.

Although we evaluated numerous factors at the prefecture level, their correlations with the suicide SMRs were small and insignificant. This result appears counterintuitive given the existing evidence, such as the association between alcohol consumption and suicide^28^ and the role of palliative care in mitigating psychological symptoms.^29^ This result may be attributable to the relationship between the suicide SMRs and prefectural‐level factors being too distant to detect potential associations. Nevertheless, cautious interpretation is required due to the potential of ecological fallacy, as the findings of this study may not represent individual‐level relationships.

This study had several limitations. First, although we examined regional variations at the prefecture level, different trends could emerge between urban and rural areas within the same prefecture. Second, we were unable to explore regional risk variations stratified by several factors such as age and sex. Third, although we examined the association between suicide and prefecture‐level factors, we were unable to obtain many potential predictors, such as lithium levels in tap water.^30^ Finally, the number of suicides observed in each prefecture was small.

In conclusion, this nationwide population‐based study found that patients with cancer living in certain prefectures, such as the Hokuriku region, had a higher risk of suicide than those living in other prefectures. This novel finding suggests that these areas should advance region‐specific countermeasures for the mental health of patients with cancer to prevent suicide.

## AUTHOR CONTRIBUTIONS

**Ken Kurisu:** Conceptualization (lead); data curation (lead); formal analysis (lead); investigation (lead); methodology (lead); software (lead); validation (lead); visualization (lead); writing – original draft (lead). **Saki Harashima:** Conceptualization (supporting); investigation (supporting); resources (lead); writing – review and editing (equal). **Maiko Fujimori:** Conceptualization (supporting); funding acquisition (lead); resources (supporting); supervision (lead); writing – review and editing (equal). **Tatsuo Akechi:** Conceptualization (supporting); writing – review and editing (equal). **Kazuhiro Yoshiuchi:** Conceptualization (supporting); writing – review and editing (equal). **Yosuke Uchitomi:** Conceptualization (supporting); writing – review and editing (equal).

## CONFLICT OF INTEREST STATEMENT

The authors declare no potential conflicts of interest.

## ETHICS STATEMENT

The study was approved by the institutional review board of the National Cancer Center Japan (approval number: 2018‐233). The requirement for informed consent was waived owing to the use of anonymous data.

## Supporting information


**Table S1.** Data sources of factors measured at the prefectural level.
**Table S2.** Descriptive statistics of the study participants.
**Table S3.** Unadjusted relative risk of suicide within 2 years following cancer diagnosis.
**Table S4.** Correlations between the standardized mortality ratio of suicide and prefecture‐level factors.
**Figure S1.** Maps of (A) raw suicide rates in patients with cancer and (B) expected suicide rates in the corresponding general population. Raw and expected suicide rates are shown as incidences per 10,000 individuals. These values were divided into three intervals with equal ranges, and each prefecture was categorized accordingly.

## Data Availability

The dataset analyzed in this study, obtained in 2022, was used with permission from the National Cancer Registry Information Desk of the National Cancer Center. This dataset cannot be shared; however, anyone can access it by sending an application to the National Cancer Registry Information Desk along with their study protocol.
