# Supplementary material for: Regional disparities in suicide among patients with cancer: A nationwide population‐based study in Japan
Source: Cancer Med. 2023 Sep 22;12(19):20052–8. doi: 10.1002/cam4.6574 (PMC10587921; doi:10.1002/cam4.6574)

**Supplementary Table 1. Data sources of factors measured at the prefectural level**

| Items | Year | Data source | URL (Last Accessed July 10, 2023) |
| --- | --- | --- | --- |
| Proportion of individuals drinking alcohol daily | 2019  2016 | Comprehensive Survey of Living Conditions | <https://www.e-stat.go.jp/stat-search/files?stat_infid=000031964440>  <https://www.e-stat.go.jp/stat-search/files?stat_infid=000031595566> |
| Proportion of individuals with a K6 score >5 or >10 | 2019  2016 | Comprehensive Survey of Living Conditions | <https://www.e-stat.go.jp/stat-search/files?stat_infid=000031964439>  <https://www.e-stat.go.jp/stat-search/files?stat_infid=000031595565> |
| Proportion of single person household or nuclear family household | 2016 | Comprehensive Survey of Living Conditions | <https://www.e-stat.go.jp/stat-search/files?stat_infid=000031592737> |
| Average income | 2016 | Cabinet Office Economic and Social Research Institute | <https://www.esri.cao.go.jp/jp/sna/data/data_list/kenmin/files/contents/main_2019.html> |
| Average household income | 2019 | National Survey of Family Income and Expenditure | <https://www.stat.go.jp/data/zenkokukakei/2019/kekka.html#kekka> |
| Reimbursement points of outpatient palliative care | 2016 | NDB Open Data | <https://www.mhlw.go.jp/stf/seisakunitsuite/bunya/0000177221_00002.html> |
| Reimbursement points of guidance for patients with cancer reimbursement | 2016 | NDB Open Data | <https://www.mhlw.go.jp/stf/seisakunitsuite/bunya/0000177221_00002.html> |
| Reimbursement points of guidance for patients with cancer to reduce psychological distress | 2016 | NDB Open Data | <https://www.mhlw.go.jp/stf/seisakunitsuite/bunya/0000177221_00002.html> |
| Reimbursement points of inpatient palliative care | 2016 | NDB Open Data | <https://www.mhlw.go.jp/stf/seisakunitsuite/bunya/0000177221_00002.html> |
| Reimbursement points of psychiatry liaison | 2016 | NDB Open Data | <https://www.mhlw.go.jp/stf/seisakunitsuite/bunya/0000177221_00002.html> |
| Reimbursement points of outpatient psychotherapy (less than 30 min) | 2016 | NDB Open Data | <https://www.mhlw.go.jp/stf/seisakunitsuite/bunya/0000177221_00002.html> |
| Reimbursement points of outpatient psychosomatic therapy | 2016 | NDB Open Data | <https://www.mhlw.go.jp/stf/seisakunitsuite/bunya/0000177221_00002.html> |
| Treatment rate of depression | 2017 | Patient Survey | <https://www.e-stat.go.jp/stat-search/files?stat_infid=000031790882> |
| Number of psychiatrists and psychosomatic physicians | 2018 | Statistics of Physicians, Dentists and Pharmacists | <https://www.e-stat.go.jp/stat-search/files?stat_infid=000031889171> |
| Unemployment rate | 2016 | Labour force surveys | <https://www.e-stat.go.jp/stat-search/files?stat_infid=000031582272> |
| Proportion of elderly aged 75 years or older | 2016 | Population Estimates | --- |
| Opioid consumption | 2015 | Guidance on the appropriate use of medical narcotics | <https://www.mhlw.go.jp/bunya/iyakuhin/yakubuturanyou/other/iryo_tekisei_guide.html> |
| Temperature | 2020 | Japan Statistical Yearbook | <https://www.stat.go.jp/data/nenkan/72nenkan/01.html> |
| Sunshine duration | 2020 | Japan Statistical Yearbook | <https://www.stat.go.jp/data/nenkan/72nenkan/01.html> |
| Incidence of cancer | 2016 | National Cancer Registry and Population Estimates | --- |

All the total values were converted into per capita values for each prefecture. Temperature and sunshine duration data were taken from the principal city in each prefecture and were averaged from 1991 to 2020.

**Supplementary Table 2. Descriptive statistics of the study participants**

|  | Number of patients (%) |
| --- | --- |
| Age (years) |  |
| 0–39 | 80,523 (3.8) |
| 40–49 | 138,776 (6.5) |
| 50–59 | 212,982 (10.0) |
| 60–69 | 516,806 (24.2) |
| 70–79 | 643,765 (30.2) |
| ≥80 | 540,650 (25.3) |
| Sex |  |
| Female | 958,518 (44.9) |
| Male | 1,174,984 (55.1) |
| Primary tumor site |  |
| Head and neck | 49,711 (2.3) |
| Esophagus | 49,850 (2.3) |
| Stomach | 246,809 (11.6) |
| Colon | 252,500 (11.8) |
| Rectum | 116,321 (5.5) |
| Liver and intrahepatic bile ducts | 75,690 (3.5) |
| Gallbladder and other biliary tract | 41,961 (2.0) |
| Pancreas | 76,305 (3.6) |
| Lung and bronchus | 232,317 (10.9) |
| Skin | 59,162 (2.8) |
| Breast | 206,354 (9.7) |
| Cervix uteri | 66,864 (3.1) |
| Corpus uteri | 31,573 (1.5) |
| Ovary | 25,033 (1.2) |
| Prostate | 171,157 (8.0) |
| Bladder | 80,621 (3.8) |
| Kidney and urinary organs | 53,828 (2.5) |
| Brain and other parts of the central nervous system | 11,426 (0.5) |
| Thyroid | 34,329 (1.6) |
| Malignant lymphoma | 64,602 (3.0) |
| Multiple myeloma | 14,479 (0.7) |
| Leukemia | 26,465 (1.2) |
| Other | 146,145 (6.9) |
| Extension of tumors |  |
| Localized | 1,086,149 (50.9) |
| Regional | 417,518 (19.6) |
| Metastatic | 335,736 (15.7) |
| Unknown/other | 294,099 (13.8) |

**Supplementary Table 3. Unadjusted relative risk of suicide within 2 years following cancer diagnosis**

| Prefecture | Relative Risk (95% Confidence Interval) |
| --- | --- |
| Hokkaido | 1.00 (Reference) |
| Aomori | 1.96 (1.24-3.09) ^*^ |
| Iwate | 0.92 (0.49-1.74) |
| Miyagi | 1.49 (0.91-2.44) |
| Akita | 1.41 (0.83-2.38) |
| Yamagata | 1.21 (0.67-2.20) |
| Fukushima | 1.36 (0.83-2.23) |
| Ibaraki | 1.16 (0.71-1.88) |
| Tochigi | 1.58 (0.96-2.60) |
| Gunma | 1.00 (0.57-1.77) |
| Saitama | 1.18 (0.80-1.73) |
| Chiba | 1.38 (0.94-2.02) |
| Tokyo | 1.55 (1.10-2.16) ^*^ |
| Kanagawa | 1.37 (0.95-1.96) |
| Niigata | 1.70 (1.13-2.57) ^*^ |
| Toyama | 1.86 (1.08-3.21) ^*^ |
| Ishikawa | 1.72 (0.96-3.07) |
| Fukui | 1.89 (0.98-3.64) |
| Yamanashi | 1.04 (0.47-2.29) |
| Nagano | 1.38 (0.82-2.33) |
| Gifu | 1.38 (0.84-2.27) |
| Shizuoka | 1.28 (0.82-2.01) |
| Aichi | 1.48 (1.01-2.16) ^*^ |
| Mie | 1.16 (0.66-2.06) |
| Shiga | 0.98 (0.48-2.00) |
| Kyoto | 1.72 (1.08-2.72) ^*^ |
| Osaka | 1.27 (0.90-1.81) |
| Hyogo | 1.23 (0.83-1.81) |
| Nara | 1.15 (0.61-2.17) |
| Wakayama | 1.20 (0.64-2.27) |
| Tottori | 0.90 (0.33-2.51) |
| Shimane | 0.82 (0.35-1.93) |
| Okayama | 1.64 (0.98-2.74) |
| Hiroshima | 1.14 (0.71-1.84) |
| Yamaguchi | 0.98 (0.52-1.84) |
| Tokushima | 1.25 (0.56-2.76) |
| Kagawa | 1.72 (0.95-3.14) |
| Ehime | 0.93 (0.49-1.75) |
| Kochi | 0.87 (0.37-2.04) |
| Fukuoka | 1.35 (0.91-2.01) |
| Saga | 1.06 (0.48-2.35) |
| Nagasaki | 1.70 (1.01-2.85) ^*^ |
| Kumamoto | 1.15 (0.65-2.03) |
| Oita | 1.88 (1.10-3.20) ^*^ |
| Miyazaki | 1.88 (1.10-3.21) ^*^ |
| Kagoshima | 0.92 (0.51-1.68) |
| Okinawa | 1.51 (0.84-2.70) |

* p <0.05

Relative risk was quantified using a Poisson regression model without adjusting for covariates.

**Supplementary Table 4. Correlations between the standardized mortality ratio of suicide and prefecture-level factors**

| Items | Correlation (95% CI) | P value |
| --- | --- | --- |
| Proportion of individuals drinking alcohol daily in 2016 | -0.003 (-0.29 to 0.29) | 0.98 |
| Proportion of individuals drinking alcohol daily in 2019 | 0.04 (-0.25 to 0.32) | 0.77 |
| Proportion of individuals with a K6 score >5 in 2016 | -0.06 (-0.35 to 0.23) | 0.68 |
| Proportion of individuals with a K6 score >10 in 2016 | -0.07 (-0.35 to 0.22) | 0.64 |
| Proportion of individuals with a K6 score >5 in 2019 | -0.11 (-0.38 to 0.19) | 0.48 |
| Proportion of individuals with a K6 score >10 in 2019 | -0.05 (-0.34 to 0.24) | 0.72 |
| Proportion of single person household | -0.18 (-0.44 to 0.12) | 0.24 |
| Proportion of nuclear family household | 0.09 (-0.21 to 0.37) | 0.55 |
| Average income | 0.09 (-0.20 to 0.37) | 0.54 |
| Average household income | 0.12 (-0.17 to 0.40) | 0.41 |
| Reimbursement points of outpatient palliative care | 0.06 (-0.32 to 0.41) | 0.77 |
| Reimbursement points of guidance for patients with cancer | 0.03 (-0.26 to 0.31) | 0.84 |
| Reimbursement points of guidance for patients with cancer to reduce psychological distress | 0.15 (-0.15 to 0.42) | 0.33 |
| Reimbursement points of inpatient palliative care | 0.02 (-0.27 to 0.31) | 0.89 |
| Reimbursement points of psychiatry liaison | 0.11 (-0.24 to 0.43) | 0.55 |
| Reimbursement points of outpatient psychotherapy (less than 30 min) | -0.05 (-0.33 to 0.24) | 0.75 |
| Reimbursement points of outpatient psychosomatic therapy | -0.10 (-0.38 to 0.19) | 0.50 |
| Treatment rate of depression | -0.01 (-0.30 to 0.28) | 0.95 |
| Number of psychiatrists and psychosomatic physicians | -0.01 (-0.30 to 0.28) | 0.95 |
| Unemployment rate | 0.10 (-0.19 to 0.38) | 0.51 |
| Proportion of elderly aged 75 years or older | -0.14 (-0.41 to 0.16) | 0.35 |
| Opioid consumption | 0.01 (-0.28 to 0.29) | 0.97 |
| Temperature | 0.01 (-0.28 to 0.30) | 0.94 |
| Sunshine duration | -0.22 (-0.48 to 0.07) | 0.14 |
| Incidence of cancer | -0.12 (-0.39 to 0.18) | 0.43 |

**Supplementary Figure 1.** Maps of (A) raw suicide rates in patients with cancer and (B) expected suicide rates in the corresponding general population. Raw and expected suicide rates are shown as incidences per 10,000 individuals. These values were divided into three intervals with equal ranges, and each prefecture was categorized accordingly.


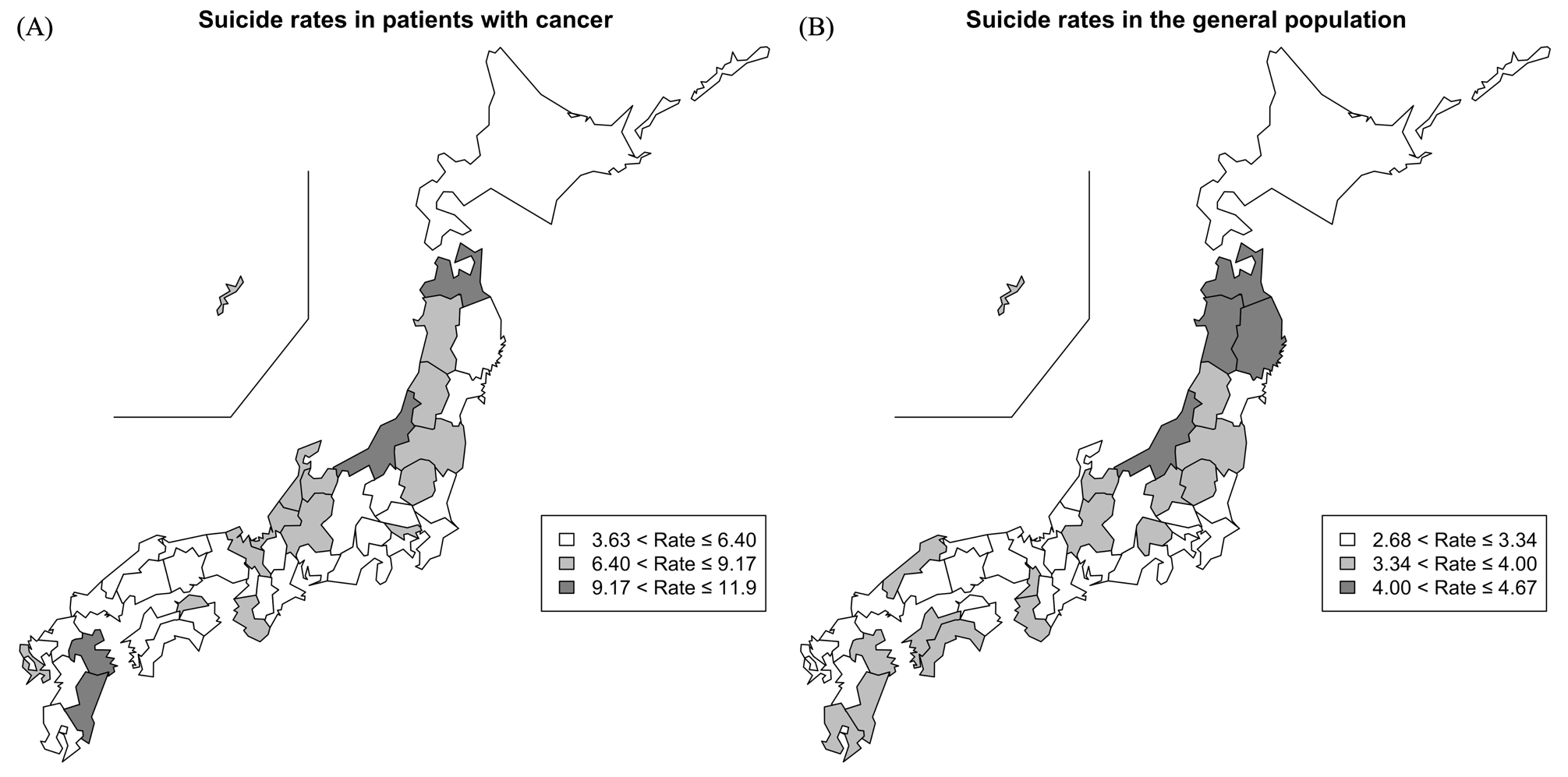

Supplement: Supplementary file 1 — Table S1. Data sources of factors measured at the prefectural level. Table S2. Descriptive statistics of the study participants. Table S3. Unadjusted relative risk of suicide within 2 years following cancer diagnosis. Table S4. Correlations between the standardized mortality ratio of suicide and prefecture‐level factors. Figure S1. Maps of (A) raw suicide rates in patients with cancer and (B) expected suicide rates in the corresponding general population. Raw and expected suicide rates are shown as incidences per 10,000 individuals. These values were divided into three intervals with equal ranges, and each prefecture was categorized accordingly. [file CAM4-12-20052-s001.docx]
